# Hyaluronic Acid Hydrogel Incorporating Dexpanthenol-Engineered Extracellular Vesicles for Accelerated Wound Closure and Mitigated Secondary Infection Risk

**DOI:** 10.3390/pharmaceutics18081003

**Published:** 2026-08-13

**Authors:** Hyeyoung Shin, Juwon Youn, Chang Kyu Lee, Seungwoon Baik, Tae-Keun Ahn, Dong Keun Han

**Affiliations:** 1 Department of Biomedical Science, CHA University, Seongnam 13488, Gyeonggi, Republic of Korea; shjyc01@naver.com (H.S.); wndnjs5485@snu.ac.kr (J.Y.); 2 College of Pharmacy, Seoul National University, Seoul 08826, Republic of Korea; 3 Department of Neurosurgery, Severance Hospital, Yonsei University College of Medicine, Seoul 03722, Republic of Korea; nscklee@yuhs.ac; 4 ORANDBIO Co., Ltd., Uiwang 16108, Gyeonggi, Republic of Korea; baiksw@orandbio.com; 5 Department of Orthopedic Surgery, CHA University, CHA Bundang Medical Center, Seongnam 13496, Gyeonggi, Republic of Korea

**Keywords:** wound healing, secondary infection prevention, hydrogel, extracellular vesicles, dexpanthenol

## Abstract

**Background:** Loss of epithelial integrity leaves the wound bed open to opportunistic bacterial colonization, and the risk of secondary infection persists for as long as the defect remains unclosed. Wound dressings must therefore provide an immediate external barrier while accelerating restoration of the skin’s own barrier. This study aims to develop and evaluate a bioactive nanotechnological platform comprising dexpanthenol (Dxp)-engineered extracellular vesicles (EVs) embedded within cross-linked hyaluronic acid hydrogels (HA@Dxp-engineered EVs) for targeted wound treatment and protection against external contaminants. **Methods:** EVs were engineered via exogenous (extrusion; Exo EV) and endogenous (co-incubation; Endo EV) strategies to encapsulate Dxp. The physicochemical properties of the HA@Dxp-engineered EV systems were characterized, and their therapeutic efficacy was validated through *in vitro* assays, including fibroblast migration and endothelial tube formation, and *in vivo* using a full-thickness excisional wound model in mice. **Results:** Both engineering strategies successfully encapsulated Dxp while preserving the structural integrity of the EVs. The HA hydrogel enabled sustained EV release and provided a physical barrier. *In vitro*, HA@Endo EVs significantly promoted fibroblast proliferation, migration, and the formation of mature capillary-like networks in HUVECs compared to controls. *In vivo*, the HA@Endo EV group demonstrated accelerated wound closure, achieving 99.88% healing by day 10, and promoted tissue remodeling with upregulated expression of COL1A1, VEGF, and HIF-1α. **Conclusions:** The HA@Endo EV system provides a dual-action strategy against secondary infection risk. It supplies an immediate physical barrier over the wound bed and simultaneously accelerates re-epithelialization, thereby shortening the interval during which the tissue remains exposed.

## 1. Introduction

The skin is the outermost organ of the body and protects internal tissues from the external environment. Upon structural damage, tissue repair typically proceeds through a highly coordinated and dynamic biological cascade comprising hemostasis, inflammation, proliferation, and remodeling [1,2]. However, this healing sequence is often disrupted in patients with underlying conditions such as aging or diabetes mellitus.

These chronic wounds frequently arrest in a prolonged, unresolved inflammatory phase characterized by excessive oxidative stress, impaired re-epithelialization, and diminished angiogenesis [3]. Most critically, the compromised epithelial integrity provides an ideal portal of entry for opportunistic pathogens, leading to recurrent secondary bacterial infections. Therefore, effectively managing such complex wounds requires therapeutic strategies that go beyond merely stimulating cellular regeneration; they must also actively establish a defensive microenvironment to mitigate the risk of secondary infectious complications.

Traditional wound dressings are insufficient for these wounds because they only cover the tissue defect [4]. Recently, biologically active dressings such as functional hydrogels have been developed to modulate the wound microenvironment [5,6]. A delivery system that promotes tissue repair and also covers the wound surface is therefore required.

Among these delivery systems, extracellular vesicles (EVs), including exosomes, have gained attention [7,8]. EVs are lipid bilayer vesicles of 30 to 150 nm secreted from most eukaryotic cells. They mediate intercellular communication. Adipose tissue-derived mesenchymal stem cell EVs (AMSC-EVs) have demonstrated substantial potential in accelerating wound closure via promoting fibroblast migration and angiogenesis [9,10,11]. As naturally derived vesicles, they do not elicit the immunogenic responses associated with whole-cell therapies [7].

However, the clinical application of free EVs is limited by their pharmacokinetic properties. When administered *in vivo*, unencapsulated EVs exhibit an extremely short circulating half-life and are often cleared within a few hours due to rapid phagocytosis and clearance by the reticuloendothelial system [7]. Consequently, maintaining a therapeutic concentration of EVs at the target site necessitates frequent and high-dose administration, which is highly inefficient and limits clinical feasibility. This challenge highlights the critical necessity of integrating EVs into a stabilizing matrix to ensure their structural preservation and sustained, localized release [3,9,12].

To resolve this delivery bottleneck and simultaneously address the risk of secondary wound contamination, embedding EVs into three-dimensional biopolymeric scaffolds, such as hyaluronic acid hydrogels (HA), represents a highly effective nanotechnological strategy. As a high-molecular-weight polymer with an interconnected 3D network, cross-linked HA hydrogels inherently possess exceptional water retention capacity and a highly porous matrix [13]. Most importantly for infection-prone wounds, the HA hydrogel serves as a conformable and durable physical barrier over the wound bed. This barrier reduces the infiltration of external pathogens and thereby lowers the risk of secondary bacterial infection [5]. Concurrently, the hydrogel network protects the structurally fragile EVs from premature enzymatic degradation and facilitates a controlled, sustained release profile, dramatically extending the therapeutic window [9,14]. MSC-derived exosomes loaded in an injectable HA hydrogel were released for over seven days under hyaluronidase treatment, and chronic wound closure was accelerated [15]. In an HA hydrogel cross-linked with a metal–organic framework, the EV cargo was maintained for more than ten days *in vivo* [11].

To further potentiate the regenerative efficacy of this nanotechnological platform, we sought to engineer the EVs by loading them with dexpanthenol (Dxp), a proven bioactive small molecule. Dxp is a stable, hydrophilic alcohol analog of pantothenic acid (vitamin B5). Upon tissue penetration, it is rapidly oxidized into pantothenic acid and incorporated into coenzyme A, a critical cofactor in cellular metabolism and lipid synthesis. Dxp has been reported to accelerate epidermal barrier repair and wound healing [16]. Its low molecular weight and hydrophilic nature make it an ideal therapeutic cargo for EV encapsulation, allowing it to reside within the aqueous core of the vesicle without destabilizing the lipid membrane.

Optimizing the incorporation of such therapeutic cargo into EVs is a critical facet of nanotechnological system design. Exosome engineering strategies are generally categorized into exogenous and endogenous methods. Exogenous approaches, such as physical extrusion, temporarily disrupt the lipid membrane of isolated EVs to force the entry of external molecules. Conversely, endogenous approaches involve supplementing the therapeutic cargo directly into the parent cell culture environment, allowing the cells to naturally package the molecule into the EVs during their biogenesis. Therefore, the two loading methods were compared in terms of loading efficiency, EV structural integrity, and biological activity.

In this study, a system consisting of Dxp-engineered EVs embedded within a cross-linked HA hydrogel (HA@Dxp-engineered EVs) was developed for cutaneous wound treatment. The physicochemical and functional characteristics of EVs engineered via exogenous (extrusion; Exo EV) and endogenous (co-incubation; Endo EV) Dxp loading were first compared. The combined effect of the hydrogel and the Dxp-loaded EVs was then evaluated. The therapeutic efficacy was assessed using *in vitro* cellular assays and an *in vivo* full-thickness excisional wound model in mice. In this work, secondary infection risk is addressed through a physical and temporal mechanism, namely barrier coverage of the exposed wound bed and accelerated restoration of the epidermal barrier, rather than through direct antimicrobial action or immunomodulation.

## 2. Materials and Methods

### 2.1. Materials

The cross-linked hyaluronic acid (HA) hydrogel with divinyl sulfone (DVS) was provided by BioPlus Co., Ltd. (Seongnam, Republic of Korea). Human umbilical vein endothelial cells (HUVECs) and Endothelial Basal Medium-2 (EBM-2) were purchased from Lonza (Basel, Switzerland). Human dermal fibroblasts (hDFs) were obtained from the American Type Culture Collection (ATCC, Manassas, VA, USA). Adipose tissue-derived mesenchymal stem cells (AD-MSCs) and Human MSC Growth Medium were provided from CEFOBio (Seoul, Republic of Korea). Dulbecco’s Modified Eagle Medium (DMEM; 1 g/L glucose), Dulbecco’s Modified Eagle Medium (DMEM; 4.5 g/L glucose), fetal bovine serum (FBS), and Dulbecco’s phosphate-buffered saline (DPBS) were obtained from Hyclone (Logan, UT, USA). Antibiotic–antimycotic solution (AA), lipophilic tracer DiO, Live/Dead staining Kit (Calcein AM/EthD-1), Pierce™ BCA Protein Assay Kit, SYBR green PCR master mix, and enhanced chemiluminescence (ECL) solution were obtained from Thermo Fisher Scientific (Waltham, MA, USA). The 500 kDa hollow fiber membrane was purchased from Repligen (Waltham, MA, USA). The Universal RNA Extraction Kit was supplied by Bioneer (Daejeon, Republic of Korea). TriZol reagent was purchased from Life Technologies (Carlsbad, CA, USA). The PrimeScript RT Reagent Kit was acquired from TaKaRa Biotechnology (Kusatsu, Japan). The Cell-count Kit (CCK-8) was obtained from Dongin LS (Seoul, Republic of Korea). Primary antibodies against CD63 were obtained from Abcam (Cambridge, MA, USA). Primary antibodies against TSG101 and Apo-A1 were supplied by Santa Cruz Biotechnology (Dallas, TX, USA). The HRP-linked secondary antibody was purchased from Cell Signaling Technology (Danvers, MA, USA). C57BL/6 mice were purchased from Orient Bio Inc. (Seongnam, Republic of Korea). Isoflurane was sourced from Piramal Critical Care Inc. (Bethlehem, PA, USA). The Hematoxylin and Eosin (H&E) staining Kit was obtained from VitroVivo Biotech (Rockville, MD, USA). Canada Balsam was procured from Duksan Pure Chemicals (Ansan, Republic of Korea).

### 2.2. Preparation and Characterization of Dxp-Engineered EVs

A cultured medium was prepared using DMEM (4.5 g/L glucose) supplemented with 30% CEFObio Medium, 10% FBS, and 1% AA. ADMSCs were seeded at a density of 1 × 10^6^ cells/well in 150 mm TC-treated dishes and cultured at 37 °C in a humidified atmosphere with 5% CO_2_. When 60% of the surface area was filled with ADMSCs, the cultured medium was replaced with DMEM (1 g/L glucose) containing 10% ultra-centrifuged FBS and 1% AA, and the cultured medium was obtained 5 times every 12 h. Then, a TFF system with a 500 kDa was used to isolate purified EVs.

To create EVs manipulated by the exogenous method, ADMSCs were first cultured under the same conditions as above, and then exosomes were separated by differential centrifugation. Dxp and 2.0 × 10^9^ EVs extracted from ADMSCs were mixed and diluted with DPBS buffer to create a 1 mM Dxp solution and then passed through 200 and 100 nm filters using an extruder, respectively. This process temporarily disrupted the lipid membrane of EVs, providing an opportunity for Dxp to penetrate. The amount of Dxp that penetrated into EVs was measured using high-performance liquid chromatography (HPLC; Thermo Fisher Scientific, MA, USA).

For engineering by the endogenous production method, ADMSCs were cultured in DMEM supplemented with exosome-depleted serum for 72 h with 1 mM Dxp added to the medium. Dxp, which entered the cell membrane by the diffusion principle, was encapsulated together with exosomes formed within the cells, so that exosomes containing Dxp could be released out of the cells. The Dxp-engineered EVs thus generated were obtained by collecting the medium 5 times at 12 h intervals and purifying them using a tangential flow filtration (TFF; Repligen, Waltham, MA, USA) system equipped with a 500 kDa hollow fiber membrane.

The morphology of EVs was visualized using a Transmission Electron Microscope (TEM; H-7600, 80 kV, Hitachi, Tokyo, Japan). The Zeta potential of the EVs was measured using a dynamic light scattering (Zetasizer Nano ZS; Malvern Panalytical, Malvern, UK).

The particle size distribution and concentration of Dxp-engineered EVs were determined by Nanoparticle Tracking Analysis (NTA) using MONO ZetaView^®^ (PMX-120, Particle Metrix, Meerbusch, Germany) system with a 488 nm scatter mode. The exosomes were diluted to 10^7^–10^8^ particles/mL using a filtered DPBS. For all samples, parameters were adjusted as follows to ensure reliable analysis: sensitivity at 80, shutter at 100, and minimum trace length at 15.

For the verification of the three types of EVs (EVs, Endo EVs, and Exo EVs), an equal amount of each sample (2 × 10^8^ particles) was loaded onto a 10% sodium dodecyl sulfate–polyacrylamide gel for electrophoresis (SDS-PAGE). Subsequently, proteins were transferred onto nitrocellulose membranes for analysis. Primary antibodies used for immunoblotting were selected against CD63, TSG101, and Apo-A1. An HRP-linked secondary antibody was used for the detection of the marker intensity. The membrane was exposed using an enhanced chemiluminescence solution (ECL) and visualized using a ChemiDoc™ XRS+ system with ImageLab software (version 6.0; Bio-Rad, Hercules, CA, USA).

### 2.3. Preparation and Characterization of HA Incorporating Dxp-Engineered EVs

To prepare the HA containing EVs, 1 × 10^9^ particles of the Dxp-engineered EVs were uniformly mixed into 100 μL of cross-linked HA. Field Emission-Scanning Electron Microscope (FE-SEM, Sigma, Carl Zeiss, Jena, Germany) analysis was performed to evaluate the influence of EV incorporation on the porous structure of the HA. The system was set up with an acceleration voltage of 5 kV and an emission current of 100 µA at different magnifications. Before observation, the films were sputter-coated with gold for 1 min. To visually confirm the dispersion of EVs within the HA, fluorescent tracking was conducted. A DiO cell-labeling solution (30 μL/mL) was added to 1 × 10^9^ particles of EVs and incubated at 37 °C for more than 1 h. After storing the mixture in a refrigerator for over 1 h, the labeled suspension was centrifuged using an Amicon Ultra-15 Centrifugal Filter Unit. The supernatant was removed, and the EVs were resuspended in a warm medium. This centrifugation step was repeated twice. The rheological properties of HA@Dxp-engineered EVs were then analyzed using a strain-controlled rheometer (ARES-G2, SN#4010-0255, TA Instruments, New Castle, DE, USA) with a frequency range of 0.1–10 Hz at 25 °C. The cumulative release of Dxp-engineered EVs from the HA was calculated based on the results of a BCA assay performed on samples collected over 8 days during incubation at 37 °C. The measurement of the released EV concentration was performed strictly following the manufacturer’s protocol (Thermo Fisher Scientific).

### 2.4. In Vitro Wound Healing Ability Analysis

hDFs were cultured in DMEM supplemented with 10% FBS, 1% AA in a humidified atmosphere at 37 °C with 5% CO_2_. The cell viability was measured using a CCK-8 assay. To assess the migration of hDFs promoted by HA@Dxp-engineered EVs, hDFs were seeded in 6-well plates at a density 2 × 10^5^ cells/well and cultured until they formed a confluent monolayer. The cell monolayer was then scratched using a sterilized 1 mL micropipette tip and washed twice with a DPBS solution to remove cell debris. Subsequently, the HA@Dxp-engineered EVs were applied using 6-well inserts. After 24 h of incubation, each well was observed using an optical microscope (CKX53, Olympus, Tokyo, Japan). The wound closure area was quantified using ImageJ software (version 1.53t; National Institutes of Health, Bethesda, MD, USA) and calculated as a percentage relative to the initial wound area.

For gene expression analysis, hDFs were cultured in 6-well plates and treated with the HA@Dxp-engineered EVs using 6-well inserts. After 24 h of incubation, an AccuPrep Universal RNA Extraction Kit was used to extract the total cellular RNA from the cells. A PrimeScript RT Reagent Kit was used for complementary DNA (cDNA) synthesis. Quantitative reverse transcription PCR (RT-qPCR; Thermo Fisher Scientific, Waltham, MA, USA) was performed using a Power SYBR Green PCR Master Mix. The 18S rRNA was utilized as a reference gene to normalize the expression levels of target genes involved in skin remodeling and angiogenesis. The sequences of the primers used are listed in Table 1.

### 2.5. In Vitro Angiogenic Ability Analysis

To evaluate the angiogenic ability of the HA@Dxp-engineered EVs, HUVECs were prepared using EBM-2 media containing 1% FBS. For the tube formation assay, 250 μL of Matrigel matrix was added to a precooled 24-well plate and incubated in a 37 °C incubator for 1 h to allow gelation. Subsequently, the prepared HUVECs were seeded onto the Matrigel layer at a density of 1.2 × 10^5^ cells/well, and the HA@Dxp-engineered EVs were applied using inserts. After 16 h of incubation, the HUVECs were stained with 4 μM Calcein AM and incubated at 37 °C for an additional 15 min. The formed capillary-like tube structures were photographed using an optical microscope equipped with a fluorescence lamp (U-RFL-T, Olympus, Tokyo, Japan) and the tube networks were quantified using ImageJ software.

### 2.6. In Vivo Evaluation

The animal experimental protocols were evaluated and approved by the Institutional Animal Care and Use Committee of CHA University (IACUC240167). Eight-week-old male C57BL/6 mice (Orient Bio Inc., Seongnam, Republic of Korea) were used. The mice were acclimatized for one week before the experiment under standard housing conditions with free access to food and water. A total of 32 mice were allocated to four groups, namely Native, Control, HA, and HA@Endo EVs (*n* = 8 per group). Mice were allocated to the groups before wound creation by arbitrary selection from the cage, and a formal randomization sequence was not generated. No animals died or were excluded during the study, and no adverse events were observed. Humane endpoints were not defined for this study. The mice were anesthetized with 2% isoflurane (Piramal Critical Care Inc., Bethlehem, PA, USA). The dorsal hair of the mice was shaved, and two full-thickness wounds were created on the dorsum of each mouse using an 8 mm circular biopsy punch. The same treatment was applied to both wounds of each animal. Wounds were not created in the Native group, which served as an intact skin control for the histological and gene expression analyses. The respective treatments were applied on days 0 and 3, and the wound healing progress was monitored for 10 days. The wound areas were photographed on days 0, 1, 3, 5, 7, and 10, and quantified using ImageJ software. The wound closure rate was calculated using the following formula: Wound closure rate (%) = (W_0_ − W_d_)/W_0_ × 100, where W_0_ is the initial wound area on day 0, and W_d_ is the wound area on day d post-treatment. The values obtained from the two wounds of each mouse were averaged, and the animal was used as the unit of analysis. Blinding of the outcome assessment was not implemented, because the hydrogel remained visually distinguishable on the wound bed. Individual animals were not identified during the wound area measurement. Survival and wound healing conditions for every group were documented throughout the experiment.

On day 10, the mice were sacrificed, and the regenerated skin tissues were harvested for further evaluation. Within each group, the animals were assigned to histological analysis (*n* = 4) and to quantitative real-time PCR (*n* = 4). For histological analysis, the tissues were fixed in 4% paraformaldehyde for 3 days and embedded in paraffin. The paraffin blocks were sectioned at a thickness of 5 µm. The sections were then deparaffinized using xylene, rehydrated through a graded ethanol series, and stained with H&E following the manufacturer’s instructions. The slides were mounted with Canada Balsam and examined under an optical microscope without blinding to group allocation.

For gene expression analysis of the regenerated tissues, total RNA was extracted from the harvested mouse skin tissues using a TRIzol reagent. Subsequently, a PrimeScript RT Reagent Kit was used for cDNA synthesis. RT-qPCR was performed using a Power SYBR Green PCR Master Mix. The 18S rRNA was utilized as a reference gene to normalize the expression levels of target genes involved in skin remodeling and angiogenesis. The sequences of the primers used are listed in Table 1.

### 2.7. Statistical Analysis

In vitro experiments were performed in at least three independent replicates. For the *in vivo* study, the animal was used as the unit of analysis. The wound closure analysis included the three wounded groups (*n* = 8 per group), and the histological and gene expression analyses were performed on four animals per group. The quantitative results were presented as means ± standard deviation (SD). * *p* < 0.05, ** *p* < 0.01, *** *p* < 0.001, and ^#^ *p* < 0.0001 indicate statistical difference, respectively. Statistically significant differences were evaluated by one-way analysis of variance (ANOVA) following Tukey’s method in GraphPad Prism 10.0 software (GraphPad Software Inc., San Diego, CA, USA).

## 3. Results and Discussion

### 3.1. Characterization of the Dxp-Engineered EVs

**Figure 1:**
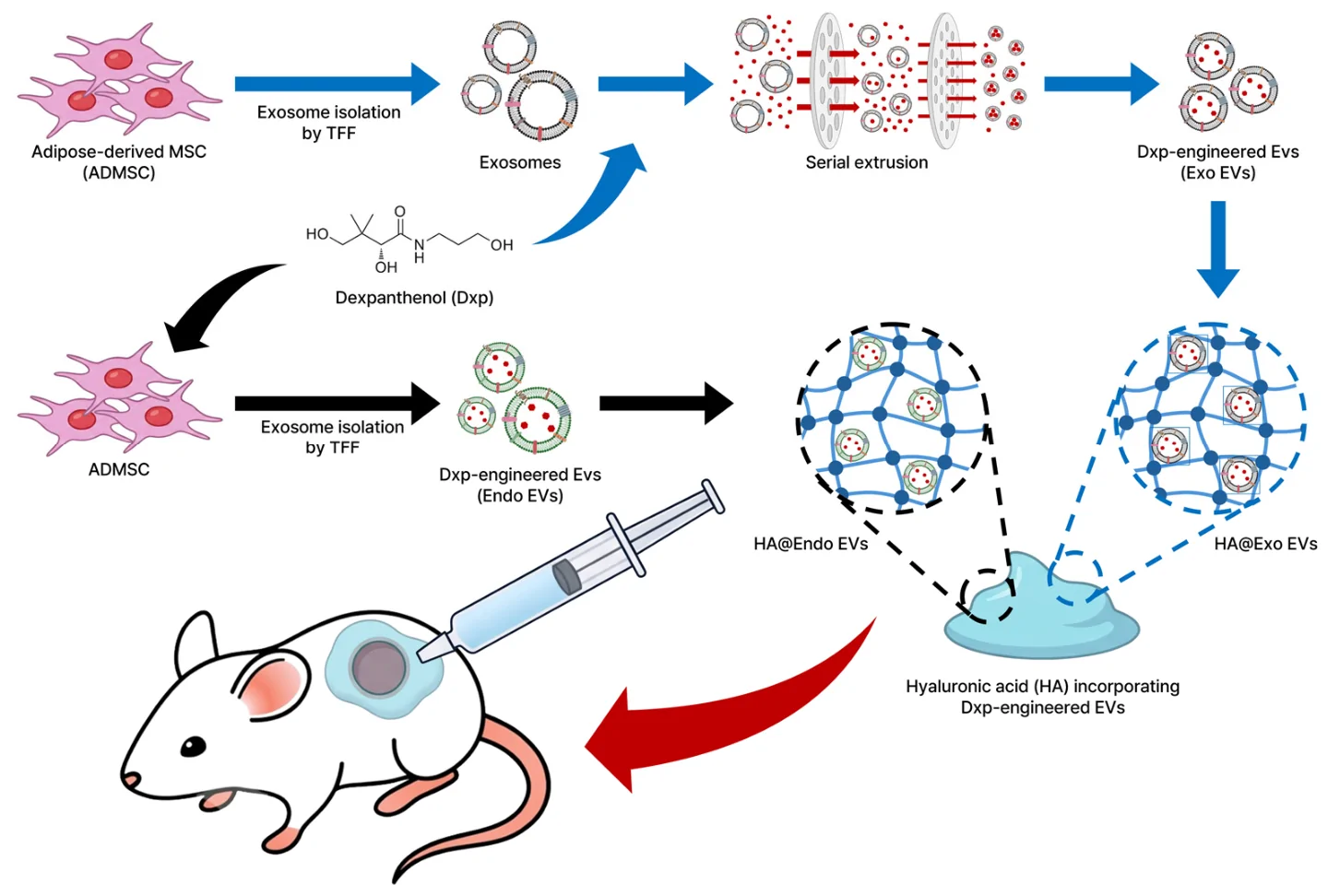
Schematic representation of the experimental workflow. Dxp-engineered EVs are prepared using exogenous or endogenous methods (Exo EV and Endo EV) and incorporated into HA hydrogels to form HA@Exo EV and HA@Endo EV. Blue arrows indicate the exogenous loading process (Exo EV), and black arrows indicate the endogenous loading process (Endo EV). These formulations are subsequently applied to a murine full-thickness skin wound model to compare their therapeutic wound healing efficacy.

Dxp-engineered EVs were fabricated using two distinct strategies. In the exogenous approach, Dxp was directly loaded into pre-isolated EVs via an extrusion process (Exo EVs). In contrast, the endogenous approach involved supplementing Dxp into ADMSC cultures, allowing for passive drug encapsulation during EV biogenesis (Endo EVs). To enhance the stability of the Dxp-engineered EVs for potential application in skin wound healing, the EVs were embedded into a cross-linked hydrogel (HA@Exo EVs and HA@Endo EVs). Their physicochemical and biological properties were characterized through *in vitro* analyses, and therapeutic efficacy was evaluated using a full-thickness excisional wound model in mice (Figure 1).

**Figure 2:**
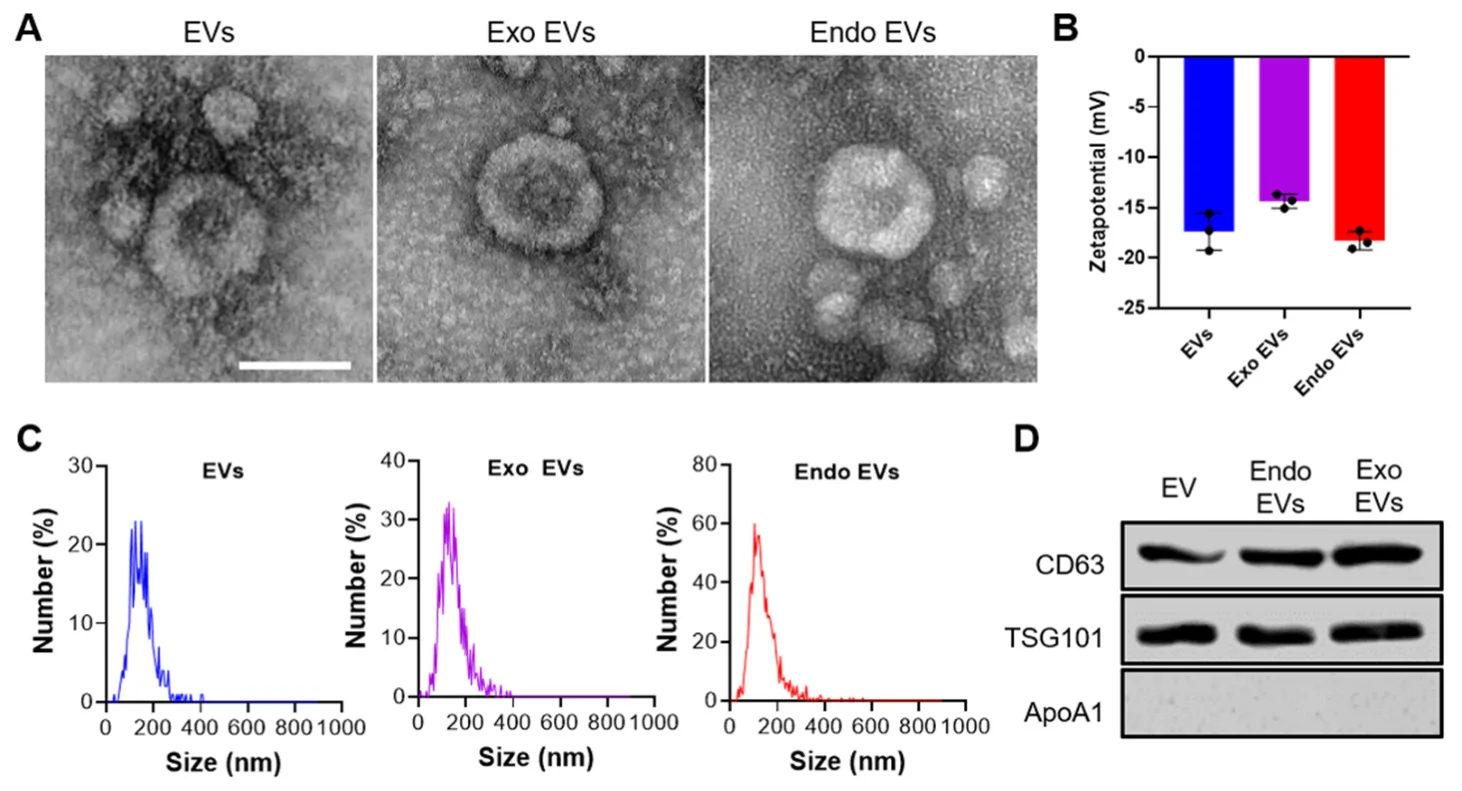
Characterization of Dxp-engineered EVs (EVs, Exo EVs, and Endo EVs). (**A**) Representative TEM images showing vesicle morphology (Scale bar = 100 nm). (**B**) Zeta potential measurements. (**C**) Size distribution profiles. (**D**) Western blot analysis of EV-specific positive markers (CD63, TSG101) and a negative marker (Apo-A1).

The morphological characteristics of the Dxp-engineered EVs were examined using TEM (Figure 2A). A characteristic phospholipid bilayer structure and spherical shape with a diameter of approximately 80 nm were observed in the EVs, Exo EVs, and Endo EVs. This size falls within the commonly reported range for EVs, which typically measure between 30 and 150 nm. The preservation of this morphology across all groups indicates that neither exogenous nor endogenous modifications disrupted the structural integrity of the EVs [17,18]. To evaluate the surface charge properties and colloidal stability, the zeta potential was analyzed (Figure 2B). The zeta potentials of the EVs, Exo EVs, and Endo EVs were −17.4, −14.4, and −18.3 mV, respectively (Figure 2B). The Exo EVs showed a lower negative charge than the native EVs and the Endo EVs. This reduction in negative charge is explained by the slightly positive surface charge measured for free Dxp (1.3 mV), which was attached to the membrane surface of Exo EVs during extrusion, leading to partial neutralization. Importantly, all groups were maintained negative charge below −14 mV, providing sufficient electrostatic repulsion to prevent aggregation and ensure long-term stability.

The concentrations of the EVs, Exo EVs, and Endo EVs were measured at 4.4 × 10^10^, 8.1 × 10^7^, and 9.1 × 10^10^ particles/mL, respectively. A significant reduction in particle yield was observed in the Exo EVs, which is likely attributable to particle loss during the extrusion process (Figure 2C). In addition to morphological and quantitative analyses, the expression of EV-associated proteins was examined (Figure 2D). CD63 and TSG101, representative exosome surface markers and extracellular matrix-associated proteins, were detected in all EV groups, whereas ApoA1, a non-exosomal marker, was not observed [19]. The consistent expression of EV-specific markers and the absence of non-EV contaminants across all groups further confirmed the identity of the isolated vesicles. Notably, the retention of marker expression after Dxp loading suggests that both loading strategies preserve the biochemical integrity of the EVs.

The Dxp loading efficiency was evaluated using HPLC (Figure S1). A single Dxp peak was detected at a retention time of approximately 3.041 min, and the peak area increased with the feeding Dxp concentration (Figure S1B,C). In the Exo EVs, the average Dxp content was measured to be 45.8 μg/mL. Given the initial Dxp concentration prior to extrusion (750 μM, equivalent to 153.9 μg/mL), approximately 29.7% of the Dxp was successfully loaded into the EVs. In contrast, for the Endo EVs, Dxp was passively encapsulated during vesicle biogenesis in the ADMSC cultures. However, due to co-packaging with various endogenous biomolecules and the absence of a defined input concentration during encapsulation, precise quantification of the Dxp content within the Endo EVs was not feasible.

These results indicate that both Exo EVs and Endo EVs possess the capacity to encapsulate Dxp while preserving their structural characteristics. In particular, the endogenous approach maintained vesicle morphology and a high particle yield, whereas the exogenous approach enabled quantifiable drug loading. This difference between the two approaches was also reflected in the surface charge. The zeta potential of the Exo EVs was less negative than that of the native EVs, whereas the Endo EVs retained a comparable value (Figure 2B). This result suggests that the membrane was rearranged or that external molecules were adsorbed during extrusion. In both groups, the EV markers CD63 and TSG101 were retained (Figure 2D), indicating that the vesicle identity was preserved after Dxp loading.

### 3.2. Characterization of the HA Incorporating Dxp-Engineered EVs

**Figure 3:**
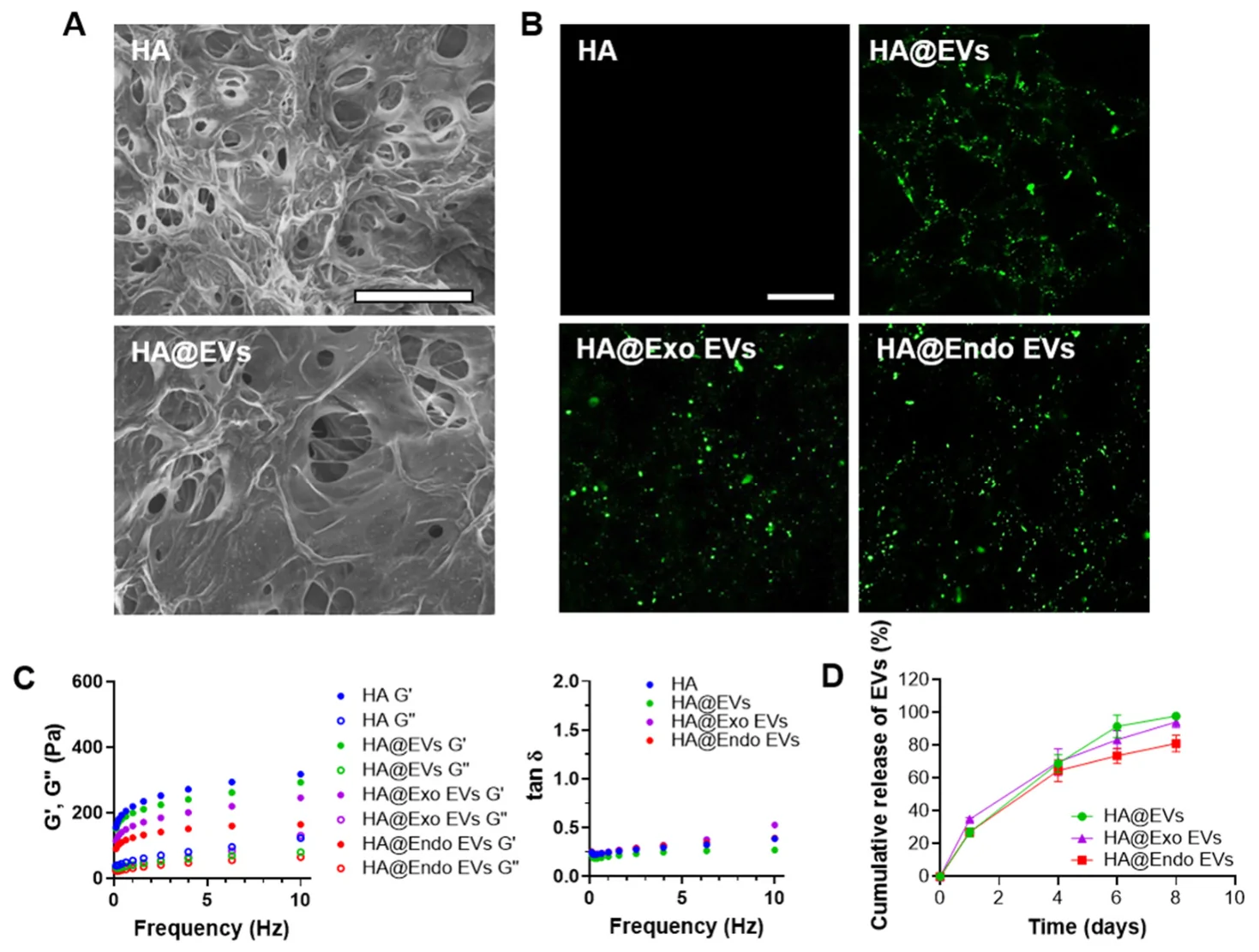
Physicochemical characterization of HA incorporating Dxp-engineered EVs. (**A**) Representative FE-SEM images of the freeze-dried empty HA and HA@EVs (Scale bar = 100 μm). (**B**) Fluorescence images of the HA incorporating DiO-labeled EVs (Scale bar = 20 μm). (**C**) Rheological properties of the experimental groups, showing the storage modulus (G′), loss modulus (G″), and viscoelastic index (tan δ) across varying frequencies. (**D**) Cumulative release profiles of EVs from HA@EVs, HA@Exo EVs, and HA@Endo EVs.

To minimize the risk of wound infection by providing a physical barrier against external contaminants and to facilitate the sustained release of therapeutic vesicles, Dxp-engineered EVs were incorporated into the hyaluronic acid hydrogel (HA). To comparatively analyze the effects of EV engineering, the experimental groups were established as empty HA, HA incorporating EVs (HA@EVs), HA incorporating Exo EVs (HA@Exo EVs), and HA incorporating Endo EVs (HA@Endo EVs). Figure 3 shows the physicochemical characterization of the groups regarding their structural morphology, distribution, rheological behavior, and release profiles. The structural morphology of freeze-dried HA and HA@EVs was examined by FE-SEM following EV incorporation (Figure 3A).

Distinct structural features attributable to the EVs were not observed under the dry-state imaging conditions. To visualize the EVs that could not be identified via FE-SEM analysis, the EVs were labeled with the lipophilic dye DiO and observed using fluorescence imaging (Figure 3B). In the HA, fluorescence signals were absent due to the lack of EVs. In contrast, strong green fluorescence was observed in the HA incorporating EVs, indicating that the labeled EVs were uniformly distributed throughout the hydrogel network without detectable aggregation. The rheological properties of the HA incorporating Dxp-engineered EVs were evaluated by measuring the storage modulus (G′) and loss modulus (G″) (Figure 3C). In all groups, G′ was consistently greater than G″, and the gap between the two moduli was observed to widen with increasing frequency. The viscoelastic index (tan δ) was maintained below 1 across all groups, indicating that elastic behavior was predominant over viscous behavior. These observations confirmed that incorporating Dxp-engineered EVs caused no significant changes in the viscoelastic properties of the hydrogel. These results indicate that the shape of the HA can be maintained at the wound bed [14,15]. Accordingly, the structural stability of the HA was preserved following the incorporation of Dxp-engineered EVs [20]. In evaluating the long-term stability and release characteristics of HA incorporating Dxp-engineered EVs, sustained release of EVs was observed for up to eight days (Figure 3D). By contrast, the half-life of naturally isolated EVs in circulation has been reported to be approximately 5.5 h. These findings indicate that Dxp-engineered EVs were effectively incorporated within the HA without compromising its structural or rheological stability, and their release was prolonged. The value of tan δ remained below 1 after the incorporation of the EVs, indicating that the cross-linked network was not disrupted by the loading. The DiO-labeled EVs were distributed throughout the hydrogel without aggregation (Figure 3B), indicating that the electrostatic repulsion between the negatively charged EVs and the HA network did not prevent uniform loading [5,6].

### 3.3. In Vitro Wound Healing Efficacy of HA Incorporating Dxp-Engineered EVs

**Figure 4:**
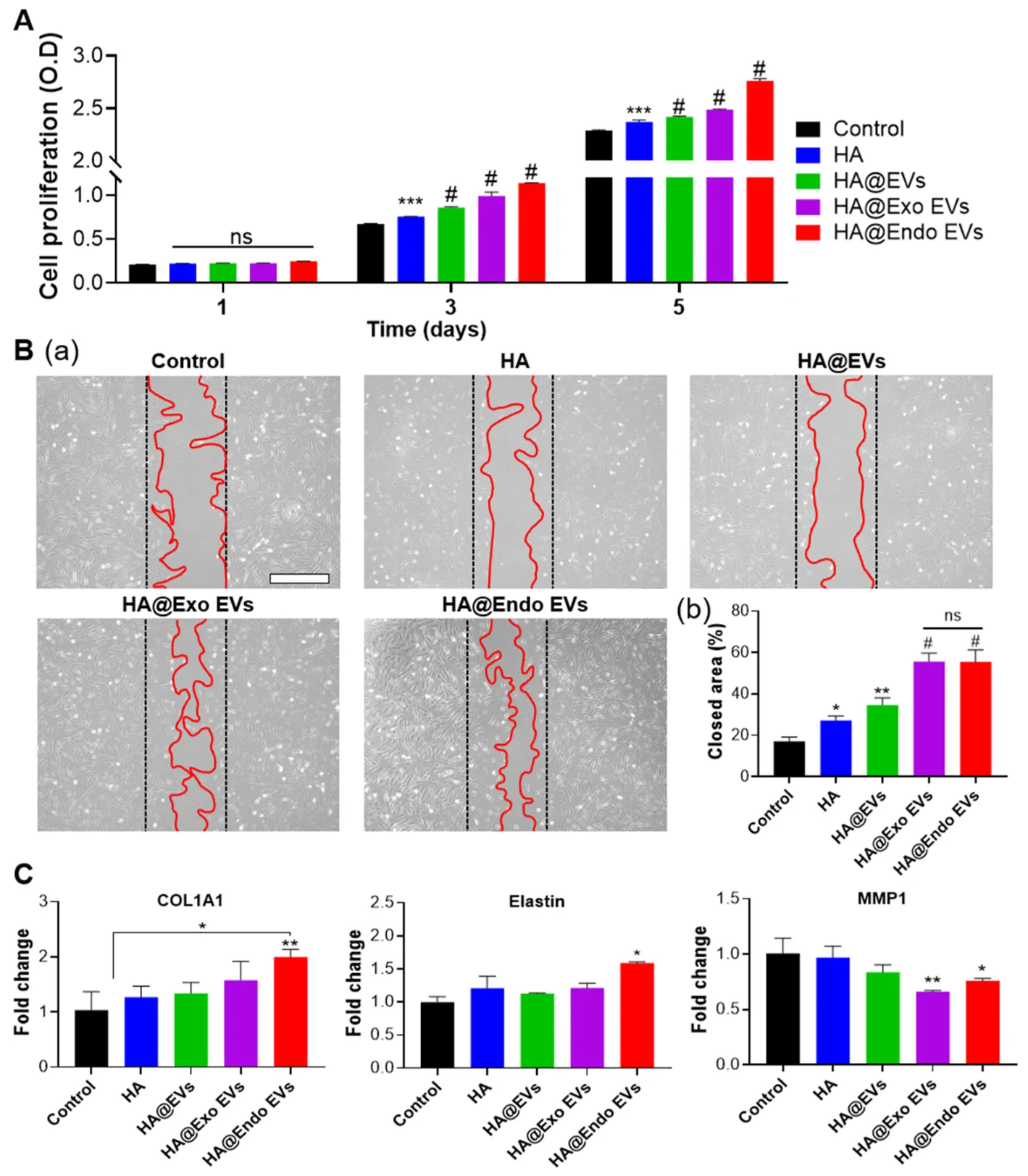
*In vitro* evaluation of the wound healing effects of HA@Dxp-engineered EVs in hDFs. (**A**) Cell proliferation over 1, 3, and 5 days. (**B**) Representative optical images (**a**) and quantitative analysis of the wound closure area (**b**) from the *in vitro* scratch assay for cell migration. Scale bar = 400 μm. (**C**) Relative mRNA expression levels of skin remodeling-related genes (COL1A1 and Elastin) and a collagen degradation-related gene (MMP-1) analyzed by qRT-PCR. * *p* < 0.05, ** *p* < 0.01, *** *p* < 0.001, and # *p* < 0.0001 indicate statistically significant differences, respectively. ns denotes no significant difference.

Figure 4 demonstrates the effect of the experimental groups on fibroblast proliferation and migration during the wound healing process. The proliferation of hDFs treated with the respective groups was evaluated over a 5-day period (Figure 4A). On day 1, no significant difference in cell growth was observed among the groups, indicating that the initial proliferative responses were comparable to the control. From day 3 onward, a stepwise increase in proliferation became evident, following the order of empty HA, HA@EVs, HA@Exo EVs, and HA@Endo EVs. By day 5, the highest proliferative activity was demonstrated in the HA@Endo EVs, with cell viability reaching approximately 120% relative to the control.

To further assess wound healing efficacy, a scratch assay was performed by generating an artificial gap in confluent hDF monolayers and subsequently treating them with the experimental groups. As shown in Figure 4Ba, cell migration into the wound area was most pronounced in the HA@Exo EVs and HA@Endo EVs compared with the control. Quantitative analysis confirmed that the wound closure ratio was significantly higher in these two groups, reaching 55.5% and 55.7%, respectively, as illustrated in Figure 4Bb. These results suggest that the Dxp-engineered EVs enhanced both fibroblast proliferation and migration, thereby contributing to wound closure. Notably, a marked difference between Exo- and Endo-loaded EVs was observed in proliferation, with the latter showing superior efficacy, whereas migration was comparably enhanced by both loading strategies. The superior performance of HA@Endo EVs compared with HA@Exo EVs indicates that endogenous loading may provide a more effective strategy for maximizing the bioactivity of EVs in wound healing applications.

**Figure 5:**
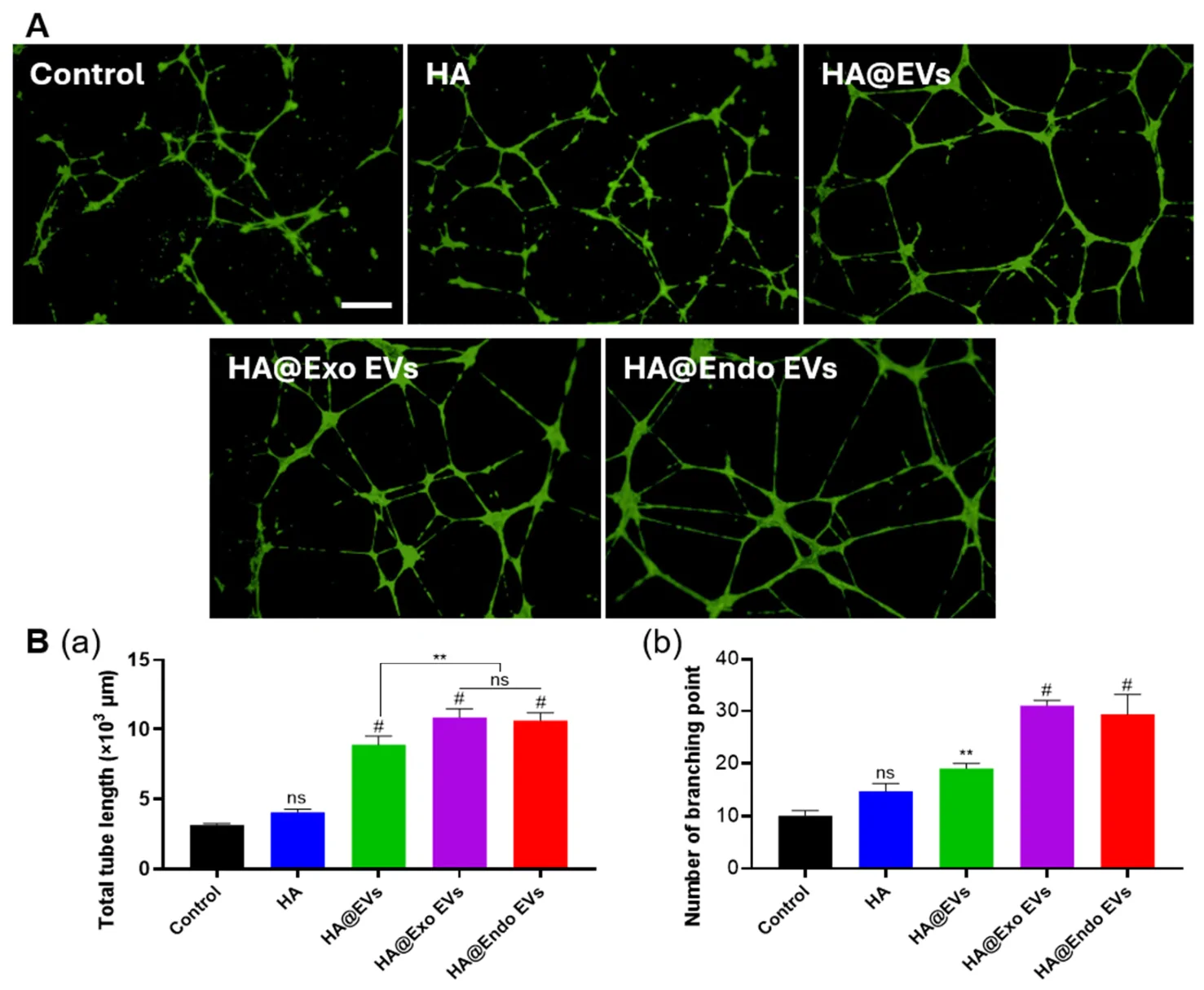
*In vitro* angiogenic effects of HA@Dxp-engineered EVs on HUVECs. (**A**) Representative fluorescence images of capillary-like tube networks formed by HUVECs stained with Calcein AM. Scale bar = 400 μm. (**B**) Quantitative analysis of total tube length (**a**) and the number of branching points (**b**). ** *p* < 0.01 and # *p* < 0.0001 indicate statistically significant differences, respectively. ns indicates no significant difference.

Further assessment of wound healing efficacy was performed by analyzing mRNA expression using RT-qPCR (Figure 5). The expression levels of COL1A1, MMP-1, and elastin were measured. When the proliferation and migration of fibroblasts are promoted and collagen biosynthesis increases, wound healing can proceed with skin tissue regeneration [21]. The most abundant fiber in the extracellular matrix (ECM) is collagen, which, in combination with elastin, provides elastic resilience to the skin. These fibers are degraded by MMPs, and the degraded fragments of elastin stimulate inflammation, proliferation, and angiogenesis [22]. When hDFs were treated with the experimental groups, the expression levels of COL1A1 and elastin were generally increased compared with the control. The relative expression levels of COL1A1 in the empty HA, HA@EVs, HA@Exo EVs, and HA@Endo EV groups were 122.6%, 129.1%, 152.3%, and 192.4%, respectively. Similarly, elastin expression exhibited an overall upward trend, reaching 121.2%, 112.7%, 121.3%, and 159.4%, respectively. In contrast, MMP-1 expression was downregulated, with relative expression levels of 96.3%, 83.0%, 65.7%, and 75.6%, respectively.

These findings suggest that HA incorporating Dxp-engineered EVs promote wound healing by enhancing fibroblast proliferation and migration and increasing collagen synthesis, and also modulating MMP-1 expression. These results indicate that wound healing was accelerated more by the HA containing Dxp-engineered EVs than by HA@EVs. Wound closure was enhanced by both Dxp-loading strategies, and the highest efficacy *in vitro* was observed in the HA@Endo EVs. The contribution of each component can also be examined from these values. The empty HA increased COL1A1 to 122.6% of the control, and the addition of unmodified EVs produced only a small further increase to 129.1%. The loading of Dxp produced the largest increase, reaching 192.4% in the HA@Endo EVs. A similar pattern was observed for elastin.

### 3.4. In Vitro Angiogenesis Effect of the HA Incorporating Dxp-Engineered EVs

To demonstrate the angiogenesis potential of the HA@Dxp-engineered EVs, a critical capability for wound healing, an *in vitro* evaluation was conducted using HUVECs (Figure 5). The fluorescence images obtained by Calcein AM staining (Figure 5A) and the corresponding quantitative analysis (Figure 5B) revealed that the engineered EVs substantially promoted the formation of capillary-like tube networks. Specifically, the total tube length and the number of branching points were evaluated to analyze the angiogenic activity. The total tube length, representing the extent of endothelial cell migration and vascular elongation, was measured. The tube lengths of the Control, HA, HA@EVs, HA@Exo EVs, and HA@Endo EVs were 3.14, 4.04, 8.88, 10.86, and 10.62, respectively. This substantial increase demonstrates that Dxp-engineered EVs effectively promote the initial sprouting and physical expansion of capillary structures. Furthermore, the number of branching points, an indicator of network complexity was analyzed. The branching points for the same groups were 10.0, 14.7, 19.0, 31.0, and 29.3, respectively. The increase in branching points indicates the formation of an interconnected capillary-like network.

The total tube length and the number of branching points were significantly higher in the HA@Dxp-engineered EVs than in the HA@EVs. These results indicate that the angiogenic activity of the EVs was enhanced by Dxp. Angiogenesis is an important process in tissue repair [23]. Blood circulation is essential for delivering oxygen, nutrients, and bioactive molecules to tissues, removing metabolic waste products, and maintaining tissue homeostasis [24]. Angiogenesis typically begins in capillaries and sprouts from parent blood vessels in a multistep process known as sprouting angiogenesis. These newly sprouted blood vessels eventually fuse with existing blood vessels or newly formed sprouts to circulate blood [23].

These observations align with previous reports demonstrating the pro-angiogenic properties of exosomes [25]. For instance, mesenchymal stem cell-derived exosomes (MSC-EVs) were confirmed to induce wound healing *in vivo* by increasing the tube formation of endothelial cells through exosomal miRNAs and improving angiogenesis via the regulation of PI3K/AKT signaling [10]. When pro-angiogenic signals, including vascular endothelial growth factor (VEGF), predominate under hypoxic conditions, endothelial cell proliferation and migration are stimulated, ultimately leading to angiogenesis [26]. Therefore, the capillary-like networks induced by HA@Dxp-engineered EVs suggest that vascularization be accelerated. In wounds at risk of infection, restored blood circulation supplies immune cells and oxygen to the wound bed [27]. The Dxp-engineered EV-loaded hydrogel is therefore considered suitable for wounds with a risk of infection. The incorporation of EVs produced the largest single increase in this assay, with the tube length rising from 4.04 in the empty HA to 8.88 in the HA@EVs. The tube length and the number of branching points were comparable between the HA@Exo EVs and the HA@Endo EVs. In contrast, the HA@Endo EVs showed higher fibroblast proliferation and higher expression of COL1A1 and elastin (Figure 4A,C). These results indicate that the two loading strategies were equivalent in promoting angiogenesis, whereas endogenous loading was more effective for fibroblast function and matrix gene expression.

### 3.5. In Vivo Evaluation of Wound Healing Effect by the HA Incorporating Dxp-Engineered EVs

**Figure 6:**
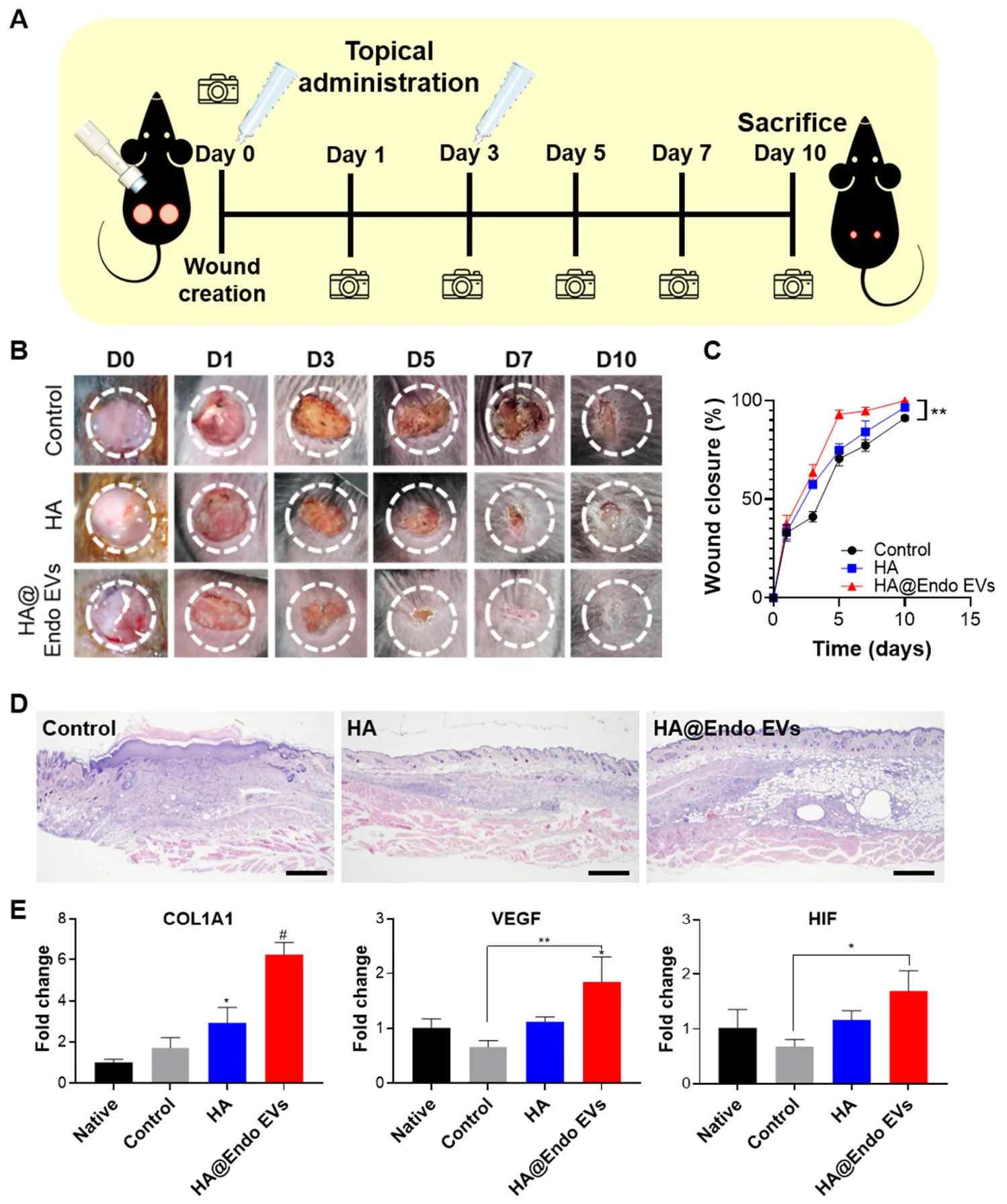
*In vivo* evaluation of the wound healing effect in a skin wound-induced mouse model over 10 days after applying the HA@Dxp-engineered EV (*n* = 8 per group for the wound closure analysis in the three wounded groups; *n* = 4 per group for the histological and gene expression analyses). (**A**) Schematic illustration of the experimental procedure for the *in vivo* skin wound-induced model. (**B**) Representative images of full-thickness skin defects in mice at days 0, 1, 3, 5, 7, and 10. (**C**) Quantitative analysis of the wound closure ratio over 10 days based on image analysis. (**D**) H&E staining images of full-thickness wounds in different groups at day 10. Scale bar: 500 μm. (**E**) qRT-qPCR was carried out to assess the relative mRNA expression levels of COL1A1, VEGF, and HIF-1α. The ‘Native’ group represents intact, uninjured normal skin. * *p* < 0.05, ** *p* < 0.01, and # *p* < 0.0001 indicate statistically significant differences.

Based on the promising cellular regeneration and angiogenic potential demonstrated in the preceding *in vitro* studies, the HA@Endo EVs was selected for the *in vivo* wound healing evaluation. To examine the therapeutic process, the animal experimental timeline and procedure were established as schematically illustrated in Figure 6A. Full-thickness skin defects were induced on day 0, and the hydrogel samples were applied immediately afterward. To ensure a sustained therapeutic effect, a second administration of the hydrogels was performed on day 3. Throughout the evaluation period of 10 days, macroscopic changes in the wounds were monitored via chronological digital photography before the mice were sacrificed on day 10 for subsequent tissue and molecular analyses.

Wound contraction was accelerated in all hydrogel-treated groups compared with the untreated control group (Figure 6B). Remarkably, the chronological evaluation highlighted a distinct acceleration in the healing kinetics among the experimental groups. While the untreated control group required the full 10 days to achieve a noticeable degree of structural tissue regeneration, the HA group reached a comparable regenerative state by day 7. In the HA@Endo EV group, the wound was largely closed and the regenerated area was observed from day 5. Quantitative analysis of the wound closure ratio over time (Figure 6C) directly supported these observations. On day 10, the degree of wound closure was 91.16%, 96.56%, and 99.88% for the control, HA, and HA@Endo EV groups, respectively. The empty HA increased the closure rate by 5.40 percentage points relative to the control, and the addition of the Dxp-engineered EVs produced a further increase of 3.32 percentage points. Importantly, alongside these rapid healing rates, macroscopic observations revealed no signs of severe inflammation or suppuration in the treated groups. This indicates that HA could act as a physical barrier against external bacterial infection at the initial stage of injury [28]. Furthermore, the rapid wound closure could contribute to reducing the duration of wound exposure to the external environment, thereby helping to minimize the risk of infection [29,30]. Consequently, this enhanced healing is primarily attributed to the combined contributions of between the sustained bioactivity of the Dxp-engineered EVs, which actively drives accelerated tissue regeneration, and the physical protective properties of the HA.

To evaluate the structural quality and remodeling of the regenerated skin, histological analysis using Hematoxylin and Eosin (H&E) staining was performed on the harvested tissues on day 10 (Figure 6D). Although the wounds were nearly closed with new epidermis in all groups, the extent of internal tissue maturation differed among the groups. In the untreated control group, the re-epithelialized tissue lacked newly formed hair follicles, and a large amount of new granulation tissue was prominently visible in the dermis, indicating that the tissue was still in the active regeneration phase. In contrast, the HA and HA@Endo EV groups successfully formed new hair follicles beneath the epidermis and contained relatively less granulation tissue in the dermis compared to the control group, suggesting they had already advanced into the tissue remodeling stage [31,32]. The HA@Endo EV group showed the most well-stratified epidermis and the most mature dermal structure.

This advanced tissue remodeling was consistently supported by molecular-level gene expression analysis at the wound site. RT-qPCR was carried out to assess the relative mRNA expression levels of key functional markers, including COL1A1 (collagen synthesis) and VEGF and HIF-1α (angiogenesis) (Figure 6E). The expression of COL1A1 was significantly upregulated in the HA@Endo EV group, as also observed *in vitro*. Collagen type 1 is a major component of the dermal matrix [33,34]. Furthermore, angiogenesis for nutrient supply and vascular tissue reinforcement was highly promoted through the increased synthesis of HIF-1α and VEGF. HIF-1α is characteristically expressed in response to hypoxia and growth factor stimulation. Subsequently, VEGF promotes angiogenesis through multiple mechanisms, including enhanced endothelial cell proliferation and survival, increased migration, and the enhanced chemotaxis and homing of bone marrow-derived vascular progenitor cells [35,36,37]. The enhanced expression of these factors efficiently restores the vascular network and accelerates overall wound healing and skin tissue regeneration [4]. These *in vivo* results indicate that skin regeneration was promoted by the HA@Endo EVs at both the structural and molecular levels. Although direct antimicrobial agents were not incorporated into the formulation, it was demonstrated that the HA@Endo EV system effectively mitigates the risk of secondary infection through a dual-action mechanism. First, the dense and highly cross-linked network structure of the HA serves as an immediate physical barrier, effectively blocking the penetration of external pathogenic bacteria into the open wound bed. Second, the combination of the hydrogel matrix and the sustained release of Dxp-engineered EVs accelerates re-epithelialization and the reconstitution of a well-stratified epidermis (Figure 6B,D). An intact stratified epidermis is the primary barrier against microbial colonization. Therefore, the period in which the wound bed is exposed to bacteria was shortened by its earlier restoration. The risk of opportunistic bacterial colonization is therefore reduced by shortening the period of exposure. Taking these observations together, the three components contributed to different aspects of repair. The HA hydrogel provided the physical environment, the EVs promoted endothelial network formation, and the Dxp increased matrix gene expression.

## 4. Conclusions

In this study, a cross-linked HA hydrogel incorporating Dxp-engineered EVs was successfully fabricated and evaluated for cutaneous wound treatment. Our findings demonstrated that incorporation of EVs into a cross-linked HA hydrogel enabled sustained EV release while improving the stability and retention of therapeutic cargo at the wound site, thereby addressing the limitations associated with the rapid clearance of free EVs. Comparative analyses further revealed that endogenous Dxp loading generated highly bioactive EVs while preserving their structural integrity, resulting in enhanced regenerative potential. The resulting HA@Endo EV platform significantly promoted fibroblast proliferation and capillary network formation, leading to accelerated tissue maturation and functional skin regeneration in a full-thickness murine wound model. In addition, rapid wound closure was achieved by combining the physical barrier of the hydrogel with the wound-healing activity of the EVs. The period of wound exposure to the external environment was therefore reduced. These results indicate that the HA@Endo EVs can be applied to cutaneous wounds with a risk of secondary infection. The present findings define the contribution of this platform to barrier restoration and tissue regeneration; the immunomodulatory activity of Dxp-engineered EVs and their efficacy under direct bacterial challenge remain to be established and will be the principal focus of our future work.

## Figures and Tables

**Table 1:** Sequence of primers used in RT-qPCR.

Gene	Primers Sequence 5′–3′
Collagen, type 1, alpha 1 (COL1A1)	FW: GGAATGAAGGGACACAGAGG, RV: AGGCTCTCCCTTAGGACCAG
Elastin	FW: TTGGAGTTGGTGCTGGTGTT RV: GCTGCTCCATATTTGGCTGC
Matrix metallopeptidase-1 (MMP-1)	FW: GCTAACCTTGATGCTATAACTACGA RV: TTTGTGCGCATGTAGAATCTG
Vascular endothelial growth factor (VEGF)	FW: ACTGGACCCTGGCTTTACTG RV: TCTGCTCCCCTTCTGTCGT
Hypoxia-inducible factor-1α (HIF-1α)	FW: TTTTTCAAGCAGTAGGAATT RV: GTGATGTAGTAGCTGCATGA

## Data Availability

The data presented in this study are available upon request from the corresponding author.

## Supplementary Materials

The following supporting information can be downloaded at https://www.mdpi.com/article/10.3390/pharmaceutics18081003/s1. Figure S1: HPLC-based quantitative analysis of Dxp loading efficiency in engineered EVs.

## Abbreviations

The following abbreviations are used in this manuscript:

AA	Antibiotic–antimycotic
AD-MSCs	Adipose tissue-derived mesenchymal stem cells
ANOVA	Analysis of variance
CCK-8	Cell-count Kit-8
cDNA	Complementary DNA
COL1A1	Collagen, type 1, alpha 1
DMEM	Dulbecco’s Modified Eagle Medium
DPBS	Dulbecco’s phosphate-buffered saline
DVS	Divinyl sulfone
Dxp	Dexpanthenol
EBM-2	Endothelial Basal Medium-2
ECL	Enhanced chemiluminescence
ECM	Extracellular matrix
EVs	Extracellular vesicles
FBS	Fetal bovine serum
FE-SEM	Field Emission-Scanning Electron Microscope
H&E	Hematoxylin and Eosin
HA	Hyaluronic acid
HIF-1α	Hypoxia-inducible factor-1α
hDFs	Human dermal fibroblasts
HPLC	High-performance liquid chromatography
HUVECs	Human umbilical vein endothelial cells
MMP-1	Matrix metallopeptidase-1
NTA	Nanoparticle Tracking Analysis
RT-qPCR	Quantitative reverse transcription PCR
SD	Standard deviation
SDS-PAGE	Sodium dodecyl sulfate–polyacrylamide gel for electrophoresis
TEM	Transmission Electron Microscope
TFF	Tangential flow filtration
VEGF	Vascular endothelial growth factor
