# Supplementary material for: Hyaluronic Acid Hydrogel Incorporating Dexpanthenol-Engineered Extracellular Vesicles for Accelerated Wound Closure and Mitigated Secondary Infection Risk
Source: Pharmaceutics. 2026 Aug 13;18(8):1003. doi: 10.3390/pharmaceutics18081003 (PMC13516435; doi:10.3390/pharmaceutics18081003)
Supplement: Supplementary file 1 [file pharmaceutics-18-01003-s001.zip › pharmaceutics-4438223-supplementary.pdf]

Hyaluronic Acid Hydrogel Incorporating Dexpanthenol-Engineered Extracellular Vesicles for Accelerated Wound Closure and Mitigated Secondary Infection Risk

Hyeyoung Shin [1,†], Juwon Youn [1,2,†], Chang Kyu Lee [3], Seungwoon Baik [4], Tae-Keun Ahn [5,*] and Dong Keun Han [4,*]

[1] Department of Biomedical Science, CHA University, Seongnam, Gyeonggi 13488, Republic of Korea
[2] College of Pharmacy, Seoul National University, Seoul 08826, Republic of Korea
[3] Spine & Spinal Cord Institute, Department of Neurosurgery, College of Medicine, Yonsei University, Seoul 03722, Republic of Korea
[4] ORANDBIO Co., Ltd., Uiwang, Gyeonggi 16108, Republic of Korea
[5] Department of Orthopedic Surgery, CHA University, CHA Bundang Medical Center, Seongnam, Gyeonggi 13496, Republic of Korea
* Correspondence: zenos@chamc.co.kr (T.-K.A); dkhan618@gmail.com (D.K.H.)
† These authors contributed equally to this work.

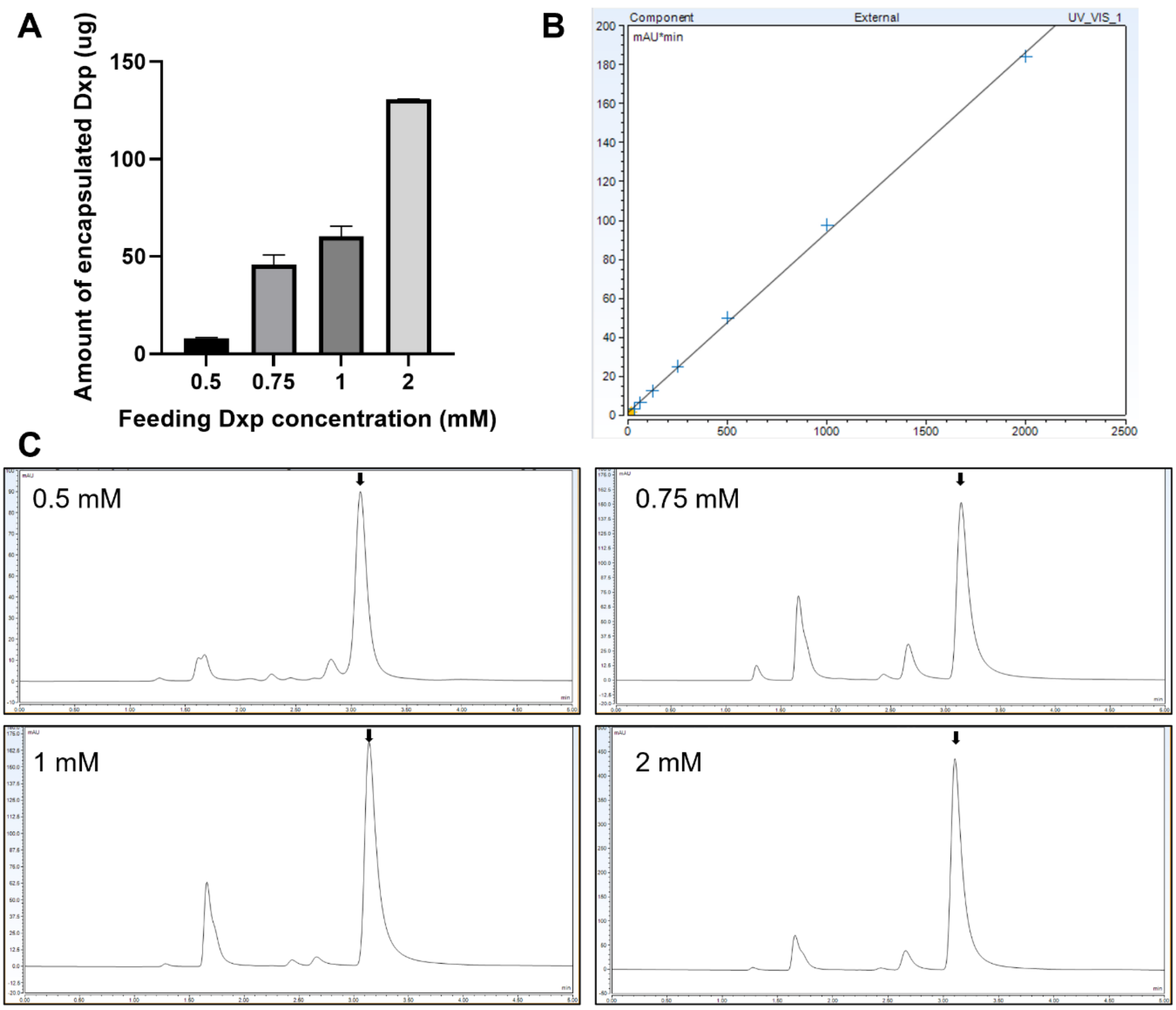

Figure S1. HPLC-based quantitative analysis of Dxp loading efficiency in engineered EVs. (A) The encapsulated Dxp content in the engineered EVs at feeding Dxp concentrations. (B) The standard calibration curve of Dxp established for the quantitative HPLC analysis (R^2 = 0.9991). (C) Representative HPLC chromatograms of the Exo EVs prepared at each feeding concentration. Arrows indicate the Dxp peak at a retention time of approximately 3.041 min.
